# Three evolutionary functions of human music: predator defense, obtaining food, preparation for war

**DOI:** 10.3389/fpsyg.2026.1858054

**Published:** 2026-07-15

**Authors:** Joseph Jordania

**Affiliations:** Faculty of Fine Arts and Music, Melbourne Conservatorium of Music, University of Melbourne, Parkville, VIC, Australia

**Keywords:** battle trance, dissonances and consonances, functions of music, music evolution, obtaining food, predator defence, war and evolution

## Abstract

Social nature of music is gradually becoming a strong scholarly consensus. In this line of thought, I want to continue discussion of the evolutionary mechanisms of several concrete functions of the human musicality. In my theoretical work on the origins and evolution of music, I have argued that music’s primary evolutionary functions were deeply connected to *survival*. Among the most important of these functions were (1) predator defense, (2) obtaining food, and (3) preparation for war. The discussion of these three functions of music is at the centre of this article. I propose that early forms of music—rhythmic vocalizations, coordinated chanting, and group-produced sound—emerged as adaptive behaviors that enhanced group cohesion and collective action in high-risk environment of African savannah. In predator defense, loud, synchronized vocal displays may have functioned as acoustic signals of group strength, deterring predators through intimidation and confusion. Also, coordinated sound-making could facilitate the aggressive scavenging scenario of obtaining food through displacement of the major predators from their kills. Finally, in preparation for war, music likely played (and still plays) a crucial role in increasing the chances of surviving (and winning) a battle by inducing emotional synchronization, reducing fear, and generating what may be termed a ‘battle trance,’ a specific altered state of consciousness enabling individual combatants to act as a unified collective.

## Introduction: sociality and music

The number of scholarly works addressing the social functions of music has grown steadily, particularly since the publication of the influential edited volume *The Origins of Music* (Wallin et al., 2000). This collection helped consolidate an emerging interdisciplinary field concerned with the evolutionary and social foundations of music. Since then, numerous scholars across disciplines—including anthropology, evolutionary biology, primatology, evolutionary psychology, sociology, and evolutionary musicology—have explored the hypothesis that music serves a range of social functions, such as social bonding, communication, credible signaling, social cohesion, identity formation, and cooperation.

Among the most influential contributors to this field is Robin Dunbar, a British biological anthropologist and specialist in primate behavior. In his seminal work *Grooming, Gossip and the Evolution of Language*, Dunbar (1996) proposed that language evolved as a mechanism for maintaining social cohesion in increasingly large groups, effectively replacing physical grooming. In his later work, he extended this framework to suggest that music may have functioned as a form of “vocal grooming,” facilitating social bonding at a group level. Similarly, Mithen (2005), in *The Singing Neanderthals*, advanced the hypothesis that music and language share a common evolutionary precursor, which he termed “musilanguage” [a concept developed by Brown (2000)]. According to Mithen (2005), early hominin vocal communication systems were holistic and emotionally expressive, serving important roles in social cohesion and coordination. Bannan (1999, 2012) has likewise emphasized the fundamentally social and collective nature of music, arguing in his works, that musical behavior is deeply embedded in cultural and social contexts. From a developmental perspective, Sandra Trehub has conducted extensive empirical research on infant musical perception (e.g., Bergeson and Trehub, 2006), demonstrating that infants are highly responsive to musical features such as pitch and rhythm. While her work is primarily descriptive, these findings have often been interpreted as supporting the view that music plays a role in early parent–infant communication and social bonding. Related ideas were articulated earlier by Dissanayake (1992), who proposed that proto-musical behaviors in mother–infant interaction formed a basis for the evolution of human musicality.

Recent theoretical developments have further elaborated the role of music in social bonding. Savage et al. (2021) argue that music evolved through a process of gene-culture coevolution as a system for enhancing group cohesion and cooperation. The genuine novelty of their contribution lies in proposing the so-called iterated Baldwin effect as a key mechanism driving this process. However, the idea that music underwent gene-culture coevolution as a system supporting group cohesion and cooperation had already been advanced by several earlier authors, including Tomlinson (2015), Killin (2016, 2017, 2018); Krasnow et al., 2017; Patel (2018), and Podlipniak (2016, 2017). In a related contribution, Mehr et al. (2021), proposed that music functions as a form of credible signaling, conveying information about coalition strength and social commitment. A complementary perspective is offered by Merker et al. (2015), who, in their discussion of common misconceptions about musical rhythm, emphasize the importance of rhythmic coordination in enabling synchronized group behavior. This capacity for entrainment is widely considered to be central to music’s role in social organization.

Other scholars have approached the question from related perspectives. Fitch (2010), in *The Evolution of Language*, explores the evolutionary relationships between music and language, suggesting that both may have played roles in social communication and coordination, while remaining cautious about strong adaptationist claims regarding music. Reybrouck (2011) emphasizes the biological and embodied dimensions of musical behavior, viewing music as a system that shapes emotional states and facilitates coordinated action.

From a developmental and interactional perspective, Trevarthen and Malloch (2009) argue that the roots of musicality lie in early communicative exchanges between caregivers and infants, particularly in rhythmic and affective synchrony. Research on cooperation by Warneken and Tomasello (2007) provides a broader framework for understanding how shared intentionality and coordinated action—capacities central to human social life—may also underpin collective musical behavior. Finally, Aniruddh (2018) in his article, where he revises his earlier position and supports an adaptive account of the origins of music-explicitly linking it to social functions. In addition, large-scale cross-cultural studies led by Savage et al. (2015) and Mehr et al. (2019) have provided empirical support for the universality of certain musical features, suggesting that music may reflect evolved tendencies toward shared rhythmic structures and socially coordinated behavior across human societies.

Taken together, this body of research highlights a range of evolutionary functions attributed to music, including social bonding, group cohesion, communication, and coordination. The volume of scholarship devoted to these topics is substantial and continues to grow, likely numbering in the thousands of publications.

However, despite this broad consensus regarding the social functions of music, relatively little attention has been paid to the role of music in concrete survival contexts central to early human evolution. In particular, the potential adaptive significance of music in situations involving immediate ecological and social pressures remains underexplored.

The present article seeks to address this gap by advancing the argument that music played a crucial role in human evolutionary history within three key domains of survival:

1. Defense against predators,
2. Facilitating food acquisition, and
3. Preparation for intergroup conflict (warfare).

By examining these domains, the article aims to provide a more functionally grounded account of the evolutionary significance of music, linking its social and emotional effects to concrete adaptive challenges faced by early human populations.

## Defending from predators

Defending from predators is historically one of the oldest and the arguably the most important sphere of survival, where music faculties could have been involved. And as we turn to the available scholarly works dedicated to this problem, we are confronted with a unique problem. On one hand, scholars believe that a valid defense strategy from predation is essential for the survival of any animal species (see Ruxton et al., 2004; Caro and Girling, 2005; Caro, 2009; Gursky and Nekaris, 2007; Fitch and Zuberbühler, 2023). At the same time, studies on the defense strategies of early humans have so far been strangely neglected. Only in June 2023, was a special interdisciplinary online conference “Defense Strategies in Early Human Evolution,” organized by the Jim Corbett International Research Centre at Grigol Robakidze University in Tbilisi, Georgia, with the participation of evolutionary biologists, paleoanthropologists, evolutionary psychologists, primatologists, neuroscientists, cognitivists, evolutionary musicologists, and conservationists, with the special volume of the conference materials published (Jordania and Wade, 2023).

The reasons for this strange neglect of this important topic in evolutionary scholarship are several. First, Darwin’s (1871) book on human evolution proposed that in human evolutionary prehistory there was no place for natural predators, as humans most likely evolved in an environment lacking dangerous predators (Darwin, 1871, p. 173). Secondly, Dart (1949, 1953) declared that early humans required no defense strategies, because they were the apex predators and ruthless killers in their ecosystem (Jones, 2008). Thirdly, with the next model, “man the hunted,” no mechanisms of defense were proposed (Brain, 1981; cf. Hart and Sussman, 2005). Instead, after the diligent study of hominin taphological remains, Brain acknowledged the immense pressure of predators on early humans, but he did not explain, how such a weak primate prey species without any serious means of defense (apart from surviving by climbing the trees) managed to live and sleep on the open savannah, gradually becoming the widest distributed mammalian species on the planet. At the same time, in the 1980s, a new important opening in this research topic appeared with the “new archaeology” paradigm (Binford, 1985; Shipman, 1986; Bunn and Kroll, 1986; Blumenschine, 1986; O'Connell et al., 1988a,b; Blumenschine and Cavallo, 1992; Dominguez-Rodrigo, 2002; Lupo and O’Connell, 2002; O’Bryan et al., 2019). The leading line of this research became the model of *aggressive scavenging* (this is when the predators are actively chased from the carcass). The main problem of this model became to explain how the weak and small early hominins were able to do that. See, for example: “microscopic analyses indicate that cut marks on some bones overlay predators’ teeth marks, showing that the hominins arrived afterward. How they got meat away from scary scavengers is anyone’s guess” (Welker, 2017, p. 149). The generally negative attitude towards scavenging in downplaying its possible role in the evolutionary past of our ancestors remains noteworthy, as people prefer to see themselves as the descendants of big game hunters, not scavengers (see Ehrenreich, 1997 on people’s overinflated attitude towards hunting and war).

When trying to answer this question, and from the perspective of the aggressive scavenging model came out the hypotheses of *aposematic defense in humans* (Jordania, 2017). We need a general explanation of this strategy. Warning display (aposematism) is an important, although often neglected defense strategy in the animal kingdom. Unlike much better known strategy of *crypsis*, which is based on the strategy of remaining invisible, silent, odourless, and fleeing as quickly as possible if discovered by a predator, *aposematism* is the completely alternative defense strategy, based on intimidating predators-to-be by remaining visible, being noisy, presenting odour, and, rather than fleeing when confronted by a predator, actively approaching and threatening the predator with body size, loud sounds, odours, and fearless behavior (Ruxton et al., 2004; Caro and Girling, 2005).

## Aposematism and sexual selection models

Aposematism and sexual selection are naturally competing strategies of survival. But there are important differences in this rivalry. Aposematism and sexual selection via male competition have many common features, and can act as complementary to each other, as the features that are useful to scare away predators, are naturally useful to scare away a rival as well. It is very different in *sexual selection via female choice*, where the features that attract female attention, are usually considered detrimental to the survival of males [as was implied by Darwin (1871) and was explicitly suggested by Zahavi in his “handicap principle.” See Zahavi (1975); but see Penn and Számadó (2020). See also Jordania (2011b)]. Whereas in the sexual selection model by female choice, the exaggerated features and sounds are declared biologically harmful (but helpful to attract mates), aposematic model, on the contrary, proposes that exactly these exaggerated features serve the function of scaring away the predators. Very interesting is the case of alternative explanations of the male peafowl train (Petrie et al., 1991; Petrie, 1994, 2021; although see Takahashi et al., 2008; Viegas, 2008). As it seems in current perspective, this is a case of *aposematic defense via intimidating the predators* (see Jordania, 2011a, 2021).

## Humans as aposematic species

Paleoanthropologist Leakey (1967) was arguably the first, who proposed that humans are aposematic, in their being unpalatable to big cats (though he did not himself employ the term *aposematic*). Leakey (1967) used his personal experience to come to this conclusion, as he witnessed on more than one occasion an aversion to human odour among lions (Leakey, 1967, p. 5). While suggesting an aposematic interpretation, Leakey’s (1967) argument is severely weakened by the fact that human flesh is not itself unpalatable to historical and contemporary predators (see, for example, Brain, 1981; Corbett, 1944; Blake, 2023). Leakey’s (1967) idea was relatively recently reviewed by Weldon (2018, p. 1) from the Smithsonian Conservation Biology Institute, who proposed that humans are possibly “*chemically aposematic*”. It is widely known among behavioral ecologists, that aposematic strategy can by no means guarantee that the animal will be immune from predators, as predators are known to eat aposematic animals with very powerful secondary defenses (for example, skunks are still eaten sometimes by very hungry dogs. See Ruxton et al., 2004). Similarly, humans can be still eaten by disabled predators (Corbett, 1944; Waltl, 2016). In books dedicated chiefly to this problem (Jordania, 2014, 2017), I argued that humans demonstrate *all the characteristics of aposematic features in every possible modality*: audio, visual, olfactory, and behavioral. Let us discuss only the audio signals, as we debate aposematism via the perspective of human musicality.

## Audio aposematic signals

Generally speaking, the popularity of the idea that human choral singing grew from animal choruses used to defend territory, is huge and still constantly growing (Hagen and Bryant, 2003; Hagen and Hammerstein, 2009; Hagen, 2022; Geissmann, 2000; Bannan, 2012; Rice, 2014:108; Mehr et al., 2021; Jordania, 2014; Harvey, 2017; Knight and Lewis, 2017; Savage et al., 2021; Leongómez et al., 2022; Nettl, 2022; Jordania and Wade, 2023). Singing is a behavior overwhelmingly distributed in arboreal and aerial ecosystems (among the tree-living and flying species). The nature and the evolutionary reasons of the appearance of these abilities is still another little-discussed problem (for example, see Podlipniak, 2023). Another type of vocal signal, the *roar*, can be a very effective in deterring predators as well, particularly as startle signals (Raine et al., 2019; Kleisner et al., 2021). And although roaring is louder than singing, and respectively, can communicate the strength more effectively than any other audio signal (Raine et al., 2019; Kleisner et al., 2021), it requires much more energy, and can damage the vocal chords, if used excessively. As a result, roar is used only for the most critical situations (typically during the actual confrontation, as a *startle* signal). In the discussion of the effectiveness of roaring, it is worth noting that collective singing (and music involving drumming), which consists of well-synchronized sequences (Hagen and Bryant, 2003) and matched pitches (Podlipniak, 2025), has an advantage over roaring in terms of long-distance signaling. It is also worth mention drumming as a possible additional behavior facilitating aggressive signaling. Drumming, although not synchronized, is observed in chimpanzees, which suggests that it may have been present in our common ancestors and could later have evolved into synchronized behavior in hominins (Fitch, 2017).

## Singing in the process of tree-to-ground transition

According to current consensus early hominins came down from the trees some 6–7 million years ago (Lieberman, 2022). And as tree-living birds and primates (including a lesser ape, gibbons) are among the most ardent singers in the animal kingdom, it would be logical to propose that our arboreal common (humans and apes) ancestor was a singer. The big questions here is why do terrestrial apes not sing? To answer this question, we need to remember that firstly, almost no terrestrial species sing (human are an exception), and secondly, many singing and noisy arboreal species (birds and monkeys) maintain silence whenever they visit (even for a short period) the ground as a cryptic strategy from potential ground predators (Jordania, 2020; Catchpole and Slater, 1995). Basically, singing is a dangerous behavior if you do not live in trees or cannot fly. So, it is natural to propose, that the ancestors of chimpanzees, gorillas and bonobos stopped singing for the same reason—maintaining cryptic cover while on the ground. In the case of non-singing arboreal orangutans, the reason for them to stop singing was most likely their *solitary lifestyle*—they do not even engage in grooming (Teboekhorst et al., 1990; Galdikas, 2005). So, in a strategically different move, early humans continued singing, therefore changing their survival strategy *from cryptic into aposematic*. This fact is crucial for understanding the human transition from arboreal to terrestrial life, as well as the subsequent coexistence of two vocal communication systems: singing and speech.

## The importance of dissonances

Allow me now to make a few professionally musicological comments. Singing in choruses can be based on *consonances* (nice, non-tense sounds) or *dissonances* (rough, tense sounds) combination of sounds or intervals. Coming from their own auditory preferences, contemporary scholars have mostly concentrated on the use of nice sounding consonances as more accepted in musical cultures (e.g., Sugimoto et al., 2010; Crespo-Bojorque and Toro, 2016). I suggested that in the context of the defense strategy *we should concentrating on dissonances* (Jordania, 2017, 2024). In highlighting this, we can recall the classic work of Helmholtz’s (1875) *On the Sensations of Tone*, where he argued that dissonance arises from beating and roughness between close frequencies. These interactions create a more agitated and attention-grabbing sound, which can be perceived as stronger or harsher than consonance. We can also see that Sethares (2005) in *Tuning, Timbre, Spectrum, Scale* develops a modern theory of *sensory dissonance*, showing how *certain* interval combinations produce greater acoustic roughness—often making them more salient and penetrating. Terhardt (1974) in his work on pitch perception and harmony also emphasizes that dissonant intervals produce more complex auditory patterns, which can enhance perceptual prominence. Huron (2006) in the book *Sweet Anticipation*, discusses how dissonance is linked to tension, arousal, and heightened attention, which can make it functionally “stronger” in emotional and behavioral terms.

In my opinion, the qualities of consonance and dissonance are innate and arise from the very nature of sound itself, which makes reference to the harmonic series particularly important in this context. Yet an important question remains: how could early humans have intentionally employed dissonance in appropriate situations unless they had already developed an understanding of consonance? Put differently, was the use of dissonance the result of deliberate choice and learned behavior, or did it emerge naturally from the intrinsic properties of dissonant sounds themselves? This is a complex issue (see Bowling and Purves, 2015; Plantinga and Trehub, 2014; McDermott et al., 2016). At the same time, comparative evidence—such as the use of dissonant vocalizations by wolves and coyotes—makes it more plausible, in my view, that the human use of dissonance originated primarily from the inherent nature of dissonant sounds rather than from conscious choice or cultural learning.

So, it seems to me, that dissonances can greatly contribute to the creation of a robust, attention-grabbing sound. For us it is very important that in the light of behavioral ecology, dissonant intervals are the most potent vocal signal for creating the loudest possible sound, the most attention-grabbing sound, and the most effective “Beau Geste” sound (when a small group wants to make an audio impression of a much larger group; Harrington, 1989; Tripovich et al., 2008). It is highly interesting in this context to note a well-known ethnomusicological fact, that the tense dissonant interval (primarily second) is widely used in the most isolated regions—in mountains of Himalaya in Tibet, Caucasus, Balkans, Alps, Ands, in island Hokkaido, Papua New Guinea central regions, and many other extremely isolated places (see the discussion of these regions with musical examples in: Jordania, 2006, 2015). Thus, dissonant harmonies, particularly the sharpest dissonant intervals, seconds, should *be historically most widespread* when a group (potentially both animal and human group) tries to warn/scare the opponent or a predator. Interestingly, in biological scholarship this useful quality of dissonances to create a potent “Beau Geste” effect was known earlier to animal experts (Harrington, 1989; Hagen and Hammerstein, 2009; Hagen and Bryant, 2003; see also Jordania, 2014, 2017).

## Rhythmic synchrony: turning defense into attack

Rhythmic synchrony was not an embellishment of early human singing—it was a weapon. Its emergence marks a decisive evolutionary rupture, transforming vocal behavior from relatively passive display into an active instrument of power (Bispham, 2006; Patel, 2008; Large and Gray, 2015; Brown, 2023). The moment voices and bodies locked into temporal unity—through synchronized singing and coordinated movement (proto-dance)—acoustic signaling crossed a threshold. It ceased to be mere expression and became collective force. This transformation did not remain defensive for long. What began as a strategy to deter predators was rapidly inverted into a strategy for confrontation. Early hominins did not simply survive alongside predators—they gradually began to challenge them. Rhythmic synchrony enabled groups to project a level of cohesion and resolve that could override interspecies hierarchies. In effect, humans hacked the predator–prey system, turning intimidation outward and in process gaining access to the most energy-rich resource in the environment: meat, acquired through aggressive scavenging and direct appropriation (Binford, 1985; Shipman, 1986; Blumenschine, 1986; O'Connell et al., 1988a,b; Lupo and O’Connell, 2002; O’Bryan et al., 2019). The comparative record exposes the radical nature of this development. Dissonant and nonlinear vocalizations—harsh, noisy, attention-grabbing, unpredictable signals—are evolutionarily ancient and taxonomically widespread. They are present in species such as coyotes and wolves (Harrington, 1989; Tripovich et al., 2008), and more broadly in alarm and aggression calls across the animal kingdom. These sounds function as what might be called “proto-dissonance”: acoustic structures that, to human perception, embody instability, tension, and threat. Morton (1977) identified the underlying principle: across species, harsh, low-frequency, noisy sounds reliably signal aggression. This is not a cultural convention but a deep biological regularity. In this sense, the roots of dissonant expression lie far back in evolutionary time.

Rhythmic synchrony, however, does not. The ability to entrain movement to a shared external pulse is almost entirely absent outside humans, appearing only in a handful of vocal-learning species such as parrots, and even there in limited or artificial contexts (Patel, 2008; Schachner et al., 2009; Merchant and Honing, 2014). This asymmetry is decisive. If dissonance is ancient and widespread, but synchrony is rare and specialized, then synchrony must be the late—and transformative—addition. The implication is unavoidable: rhythmic synchrony did not simply enhance existing vocal behavior; it redefined its function. The power of acoustic intimidation scales predictably—from individual to group, from unison to harmony, from consonance to dissonance. But synchrony introduces something categorically new: visible and audible unity. A synchronized group does not merely sound larger—it signals that it can act as one. It advertises coordination, shared intent, and the capacity for collective violence (Hagen and Bryant, 2003; Johnson and Cloonan, 2009; Jordania, 2009, 2022). All singing can intimidate. But rhythmically synchronized group singing in dissonant harmony is not just more effective—it is qualitatively different. It is the acoustic signature of a coalition ready to act. In evolutionary terms, it may have been one of humanity’s first true force multipliers. British naturalist and hunter Jim Corbett was arguably the first who wrote about the use of group singing as avoidance strategy from the attack of a man-eating tiger (Corbett, 1944, story “Chowgarh Tigers”); African Pygmies also routinely use singing when going through the jungle, or, when they believe there is a danger of the attack from leopards at night (Turnbull, 1961, p. 58; Knight and Lewis, 2017).

## The battle trance: the creation of the war machine

What follows is not a marginal refinement of human behavior but a redefinition of it. Music, in its rhythmic and collective form, did not evolve for beauty, expression, or even social bonding. It evolved as a technology of transformation—a mechanism for manufacturing a specific kind of human: the war-ready collective organism.

Rhythmic synchrony does not merely coordinate action; it rewires it. When voices and bodies align in time, they generate more than sound—they induce a mass neurocognitive shift. Through entrainment (McNeill, 1995), synchronous singing, chanting, and movement push participants into an altered state of consciousness: battle trance (Jordania, 2011a). This is not metaphor. It is a functional state, repeatedly documented yet still systematically underestimated (Wade, 2016; Gibson, 2011; Gray, 1959; Junger, 2010; Hedges, 2002; Kartomi, 2023).

In this state, the normal human cognitive configuration breaks down.

Fear—the most basic survival mechanism—is switched off. Combat accounts consistently describe a condition that can only be called *aphobia*: the absence of fear under extreme threat (Marshall, 1947; Keegan, 1976; Grossman, 2004; Jordania, 2024; Walker, 2013). Pain, likewise, is suspended. Severe injuries register weakly or not at all, overridden by stress-induced analgesia (Beecher, 1946; Horowitz, 1976). Memory fragments or disappears entirely; participants often cannot recall their own actions, as if the events belonged to someone else (Holmes, 1985; Grossman, 2004). Rational thought dissolves. Deliberation is replaced by automaticity—fast, reflexive, and unhesitating. At the same time, the boundaries of the self disintegrate. Individual identity gives way to group identity. The “I” is replaced by the “WE,” not as an idea, but as a lived neurocognitive reality (Zimbardo, 1969). Action becomes synchronized not because it is planned, but because it is embodied. In extreme cases, individuals exhibit sudden surges of strength—I would propose to call it the “superman effect”—suggesting that ordinary physiological limits are, at least temporarily, bypassed. Taken together, these features describe not an enhanced individual, but a different entity altogether. Battle trance does not produce better humans; it produces a collective system optimized for confrontation.

And this system is morally double-edged. Internally, it generates extreme altruism—self-sacrifice without hesitation. Externally, it can generate extreme aggression—violence without restraint, including against noncombatants (Atran, 2010; Jordania, 2011a). The same mechanism that binds the group dissolves the moral status of the outsider. This is not a flaw of the system; it is part of its design.

Crucially, this state can be triggered instantly in moments of crisis, but it can also be engineered. Military drill, rhythmic chanting, coordinated movement—these are not traditions or rituals in any trivial sense. They are induction protocols. From ancient war dances to modern boot camps, the method is the same: impose synchrony, amplify rhythm, and dissolve individuality (McNeill, 1995; Ehrenreich, 1997; Jordania, 2011a, 2024).

When large groups achieve precise synchrony, the effect is not merely psychological but perceptual. The group ceases to appear as a collection of individuals and instead manifests as a single, unified entity—a “super-organism” projecting scale, cohesion, and inevitability. This is intimidation at its most advanced: not noise, but organized force made visible and audible at once.

From a neurocognitive perspective, battle trance can be understood as a coordinated reconfiguration of brain and body systems: fear circuits suppressed (amygdala modulation), defensive aggression facilitated (periaqueductal gray), cognition narrowed and present-focused, perception constrained, pain inhibited, energy mobilized, and identity fused into the group. The result is fast, synchronized, high-risk action with minimal internal resistance.

The implication is unavoidable and deeply unsettling. What we call “music” in its rhythmic, collective form may be rooted not in aesthetics but in combat. It is a technology for turning groups into weapons (See also Pieslak, 2009; Johnson and Cloonan, 2009).

In this light, early humans did not simply develop better defense and attack tools—they became the tool. Through rhythm, they acquired the capacity to act as coordinated, self-sacrificial, and terrifyingly effective coalitions. Battle trance was not a byproduct of human evolution. It was one of its engines.

## Discussion: three survival mechanisms in historical perspective

At its core, the argument of this article is disarmingly simple: all three survival mechanisms discussed here are forms of organized conflict. First, fighting predators to avoid being killed and eaten. Second, fighting those same predators to take their food. Third, fighting other human groups for control of resources. What changes across these stages is not the presence of conflict, but its direction, scale, and target.

I propose that these three functions emerged in a clear evolutionary sequence.

The first and most urgent stage was direct confrontation with predators. From the moment early hominins descended onto predator-rich African landscapes, survival depended on deterring attack. Over millions of years, this pressure led to the gradual refinement of a powerful multisensory defense system—what I have termed *AVOID* (“audio, visual, olfactory intimidating display”; Jordania, 2017). This was not a marginal adaptation but a life-or-death solution to a constant existential threat.

The second stage followed as a functional reversal. Once sufficiently refined, the same defensive system was turned outward. Hominins began not merely to repel predators, but to confront them and appropriate their kills. In this shift from defense to offense, the AVOID system became a tool of aggressive scavenging. Rhythmic unity likely played a decisive role here, amplifying the perceived size, coordination, and resolve of the group. This is the context in which key aspects of human morphology and behavior were shaped: persistence in open savannahs, tracking predators, locating kills via scavenger birds, and confronting dominant carnivores such as lions. The archaeological and biogeographical record suggests a long association between hominins and large predators, particularly lions, which early humans appear to have followed across landscapes (Jordania, 2014; Barnett et al., 2006; Somerville, 2019; Willems and van Schaik, 2017). Even today, this strategy survives in attenuated form among groups such as the Hadza and Dorobo of East Africa (O'Connell et al., 1988a,b; Johnsingh, 2023).

The third stage marks a profound redirection. Once the problems of predator defense and food acquisition were sufficiently stabilized, the same mechanisms—AVOID displays, rhythmic synchrony, and battle trance—were turned inward, toward other human groups. What began as interspecies confrontation became intraspecies conflict. From small-scale group clashes to organized warfare among tribes, states, and ideologies, this stage defines the comparatively recent but overwhelmingly dominant chapter of human history.

This perspective also reframes a long-standing claim in evolutionary musicology. Miller (2000) famously argued that music offers no clear survival benefit in terms of finding food, avoiding predators, or resisting parasites. From the standpoint developed here, that claim is no longer sustainable. On the contrary, early music—understood as coordinated, rhythmic, collective vocalization—was directly implicated in at least two of these fundamental survival functions: predator deterrence and facilitating food acquisition. Far from being biologically marginal, music may have been instrumental in solving precisely the problems Miller considered central.

It is tempting to argue that these functions are now obsolete. In part, they are. Most humans no longer face daily predation or need to compete with large carnivores for food. Yet the underlying mechanisms have not disappeared. In regions where humans still live alongside dangerous wildlife, the inherited aversion of predators to coordinated human displays remains a critical protective factor—an outcome of a long evolutionary “arms race.” Similarly, practices resembling aggressive scavenging persist in some traditional societies (Johnsingh, 2023). And perhaps most tellingly, modern military institutions continue to rely on rhythmically synchronized movement, chanting, and drill to induce the same altered state described here as battle trance.

What has changed is not the mechanism, but its context. The original survival functions of music are now obscured beneath its modern roles in art and entertainment. Yet this is a surface transformation. Beneath it, the older biological functions remain intact and, in certain contexts, fully active. Music, in this view, is a deeply rooted biological technology—one that helped early humans survive, compete, and ultimately dominate. Its evolutionary role has been underestimated not because it was minor, but because it has been too successfully integrated into domains where its original function is no longer visible.

## Data Availability

The original contributions presented in the study are included in the article/supplementary material, further inquiries can be directed to the corresponding author.
